# Ovarian Cysts in Negresses

**Published:** 1897-10

**Authors:** 


### OVARIAN CYSTS IN NEGRESSES.

WASHINGTON, D. C., September 6, 1897.

*Editor Atlanta Medical and Surgical Journal:*

The undersigned requests information in reference to multilocular ovarian cysts in negresses. In his experience they are very rare, while parovarian, papillomatous, dermoid, and broad ligament cysts are occasionally seen. Letters of inquiry have been addressed to nearly all the members of the Southern Surgical and Gynecological Association, who should have by far the most experience in treating these cases. The answers thus far received, for the most part, agree with the experience of the writer. It is earnestly requested that surgeons generally will aid in the collection of enough information to be of value, as there seems to be no authentic record of cases, nor even of an effort to investigate the subject. Gentlemen having operated for ovarian tumors occurring in negresses will please state the number of cases, age, size of tumor, and the color of the patient.

Address I. S. Stone, M.D., 1449 Rhode Island Ave. N. W., Washington, D. C.

